# Class B Scavenger Receptor CD36 as a Potential Therapeutic Target in Inflammation Induced by Danger-Associated Molecular Patterns

**DOI:** 10.3390/cells13231992

**Published:** 2024-12-03

**Authors:** Irina N. Baranova, Alexander V. Bocharov, Tatyana G. Vishnyakova, Zhigang Chen, Yunbo Ke, Anna A. Birukova, Peter S. T. Yuen, Takayuki Tsuji, Robert A. Star, Konstantin G. Birukov, Amy P. Patterson, Thomas L. Eggerman

**Affiliations:** 1 Department of Laboratory Medicine, Clinical Center, National Institutes of Health, Bethesda, MD 20892, USA; abocharov@cc.nih.gov (A.V.B.); vishnyat@cc.nih.gov (T.G.V.); chenz2@cc.nih.gov (Z.C.); amy.patterson@nih.gov (A.P.P.); eggermant@extra.niddk.nih.gov (T.L.E.); 2 Department of Anesthesiology, University of Maryland School of Medicine, Baltimore, MD 21201, USA; yke@som.umaryland.edu (Y.K.); kbirukov@som.umaryland.edu (K.G.B.); 3 Department of Medicine, University of Maryland School of Medicine, Baltimore, MD 21201, USA; abirukova@som.umaryland.edu; 4 Renal Diagnostics and Therapeutics Unit, National Institute of Diabetes and Digestive and Kidney Diseases, National Institutes of Health, Bethesda, MD 20892, USA; petery@intra.niddk.nih.gov (P.S.T.Y.); tsu@hmedc.or.jp (T.T.); starr@niddk.nih.gov (R.A.S.); 5 Office of the Director, Division of Program Coordination, Planning and Strategic Initiatives, National Institutes of Health, Bethesda, MD 20892, USA; 6 Division of Diabetes, Endocrinology and Metabolic Diseases, National Institute of Diabetes and Digestive and Kidney Diseases, National Institutes of Health, Bethesda, MD 20892, USA

**Keywords:** CD36, DAMPs, HMGB1, histones, SAA, heat shock proteins, oxPAPC, inflammatory markers

## Abstract

The class B scavenger receptor CD36 is known to bind and mediate the transport of lipid-related ligands and it functions as a pattern recognition receptor (PRR) for a variety of pathogens, including bacteria and viruses. In this study, we assessed CD36’s role as a PRR mediating pro-inflammatory effects of several known Danger-Associated Molecular Patterns (DAMPs) used either as a single preparation or as a combination of DAMPs in the form of total cell/skeletal muscle tissue lysates. Our data demonstrated that multiple DAMPs, including HMGB1, HSPs, histone H3, SAA, and oxPAPC, as well as cell/tissue lysate preparations, induced substantially higher (~7–10-fold) IL-8 cytokine responses in HEK293 cells overexpressing CD36 compared to control WT cells. At the same time, DAMP-induced secretion of IL-6 in bone marrow-derived macrophages (BMDM) from CD36−/− mice was markedly (~2–3 times) reduced, as compared to macrophages from normal mice. Synthetic amphipathic helical peptides (SAHPs), known CD36 ligands, efficiently blocked CD36-dependent inflammatory responses induced by both cell and tissue lysates, HMGB1 and histone H3 in CD36+ cells. IP injection of total cellular lysate preparation induced inflammatory responses that were assessed by the expression of liver and lung pro-inflammatory markers, including IL-6, TNF-α, CD68, and CXCL1, and was reduced by ~50% in CD36-deficient mice compared to normal mice. Our findings demonstrate that CD36 is a PRR contributing to the innate immune response via mediating DAMP-induced inflammatory signaling and highlight the importance of this receptor as a potential therapeutic target in DAMP-associated inflammatory conditions.

## 1. Introduction

CD36 is a multifunctional transmembrane glycoprotein receptor that belongs to the class B scavenger receptor family, alongside 3 other members: LIMP-2 (lysosomal integral membrane protein–2), CLA-1 (CD36 and LIMP-2 analogous, or SR-BI, scavenger receptor BI), and its splicing variant CLA-2 (or SR-BII). CD36 is primarily expressed on the surface of various innate and adaptive immune cells, including macrophages, monocytes, dendritic cells, and subsets of T and B cells. It is also found in many non-immune cells, such as adipocytes, platelets, hepatocytes, pancreatic β-cells, certain epithelial cells, and microvascular endothelial cells [1]. The receptor can bind a wide range of ligands, including oxidized low-density lipoprotein (oxLDL), thrombospondin-1 [2], oxidized phospholipids, fibrillar Aβ amyloid peptides, serum amyloid A [3], and long-chain fatty acids [4], and interacts with *Plasmodium falciparum*-infected erythrocytes [5], bacterial components [6], and apoptotic cells. As a result, it plays an important role in glucose and lipid metabolism, inflammation, immune response, thrombosis, angiogenesis and fibrosis [1]. As a scavenger receptor and a pattern recognition receptor (PRR), CD36 recognizes and clears specific pathogen-associated molecular patterns (PAMPs), presented by pathogens or pathogen-infected cells. It functions as an endocytic receptor for bacteria [7,8], and a signaling receptor for multiple PAMPs, including bacterial products like LPS [6], LTA [9], GroEL [6], and β-glucan from fungal cell walls [10]. Moreover, recent studies have identified CD36 as a co-receptor for HCV E1 protein, facilitating viral attachment and contributing to HCV replication within the host cells [11].

Following trauma, severe infection, or stress, various intracellularly sequestered endogenous molecules with physiological functions within the cell are released into the extracellular space, triggering a non-infectious inflammatory response known as sterile inflammation. These molecules, termed Damage-Associated Molecular Patterns (DAMPs), activate innate immunity by interacting with PRRs, particularly toll-like receptors (TLRs) [12] and scavenger receptors [13]. While DAMPs are essential for the host’s defense, they can also induce pathological inflammatory responses. Several DAMPs, including high-mobility group box 1 protein (HMGB1), heat shock proteins (HSPs), and S100 proteins, are increased in inflammatory diseases and exhibit pathogenic roles [14]. Multiple studies indicate that various damaged tissues, resulting from crush injuries, chemical insults, burns, cold exposure, oxygen deprivation, radiation, tumors, and other factors can trigger sterile inflammation driven by the DAMPs [15]. CD36, like other scavenger receptors, recognizes various endogenously produced ligands, including apoptotic cells [2,16,17], oxidatively modified lipoproteins [18,19], amyloid-forming peptides, and glycated proteins [20,21]. It has been identified as a critical regulator of sterile inflammation, particularly by mediating NLRP3 inflammasome activation [22].

DAMPs include a diverse group of host-derived molecules from multiple sources, including extracellular proteins, such as tenascin C and biglycan, and intracellular proteins, like histones, HMGB1, HSPs, and plasma proteins, such as Gc-globulin, fibrinogen, and serum amyloid A [23,24,25,26,27,28,29]. HMGB1, the most well-characterized DAMP, is a nuclear protein that regulates gene transcription under normal conditions. In response to cellular damage, HMGB1 can be actively secreted by activated or stressed immune and non-immune cells or passively leak from dying cells. Once released, HMGB1 activates the innate immune response through interactions with its known receptors, including the most studied ones, TLRs and RAGE (receptor for advanced glycation end-products) [30]. A recent study [31] demonstrated HMGB1-induced Erk and Akt signaling pathways activation in bone marrow-derived macrophages from wild-type mice but not from CD36-deficient mice, suggesting a CD36-dependent mechanism for its effects.

Skeletal muscle crush injury is a common tissue trauma in humans that can cause significant morbidity in both civilian and military populations. Natural disasters, like tornadoes and earthquakes, etc., can cause mass casualties, with about 40% of victims trapped in the rubble, suffering from crush syndrome and other severe injuries. Limb compression for an extended time sustained during military conflicts also frequently results in crush injuries to skeletal muscle, which is usually associated with muscle tissue breakdown followed by the leakage of myocyte contents into the plasma. The massive release of intracellular DAMPs from damaged tissue into the circulation activates immunity via engaging PRRs and initiates systemic inflammatory responses. Several animal models of skeletal muscle-crush injury are used to study the pathophysiology of acute muscle inflammation and to investigate potential therapies. These models are designed to closely simulate a real-world crush injury and mimic human clinical presentation. Although open and closed models of skeletal muscle crush injury in small animals are available, they are limited by their need for surgically isolating the muscle or by potentially fracturing the fibula, respectively.

In this study, we investigated a CD36 role in mediating DAMP-induced inflammatory response in vivo, in a simplified model of a crush injury, achieved by the IP injection of total cell homogenate, and in vitro via cell treatment with cell/mouse skeletal lysates, used as a prototype mix of DAMPs. The results of this study demonstrate that pro-inflammatory responses induced by the individual DAMPs, as well as by their mix, appeared to be CD36-dependent, further supporting a CD36 role in DAMP-induced inflammation and suggesting this scavenger receptor is a promising target for therapeutic intervention.

## 2. Materials and Methods

### 2.1. Reagents

LPS (*Escherichia coli* O111:B4) and recombinant mouse macrophage colony-stimulating factor (M-CSF) were purchased from Sigma-Aldrich (St. Louis, MO, USA). Human recombinant HMGB1 was from Sino Biological, human recombinant apo-SAA was from PeproTech (Rocky Hill, NJ, USA) and histone H3 from calf thymus was from Roche (Mannheim, Germany). Recombinant human HSP60 and HSP70 were from Novus Biologicals. PAPC (1-Palmitoyl-2-arachdonyl-phosphatidylcholine), obtained from Avanti Polar Lipids (Alabaster, AL, USA), was oxidized by exposure of dry lipid to air as previously described [32,33]).

All media, sera, antibiotics, and all reagents used for RNA isolation, reverse transcription, and real-time PCR as well as enzyme-linked immunosorbent assay (ELISA) kits for quantifying mouse IL-6 and human IL-8 were purchased from Thermo Fisher Scientific (Waltham, MA, USA).

The peptides were synthesized by a solid-phase procedure as previously reported [34,35]. Peptide sequences were described in a previous report [36,37]. The MAPK inhibitors PD98059, SB202190, and SP600125 were from EMD Biosciences (San Diego, CA, USA), and PP2, a selective inhibitor of the Src family of protein tyrosine kinases, was from Sigma-Aldrich (St. Louis, MO, USA). Protease and phosphatase inhibitor cocktails were from Thermo Fisher Scientific.

### 2.2. Animals

All animal care and treatment procedures were approved by the University of Maryland Animal Care and Use Committee (IACUC protocol # 1022001). Animals were handled according to the National Institutes of Health’s Guide for the Care and Use of Laboratory Animals [38]. Male C57BL/J mice (8 to 10 weeks old), with an average weight of 20 to 25 g, were obtained from Jackson Laboratory (Bar Harbor, ME, USA). CD36 KO mice (C57BL/6 background) were kindly provided by Dr. Kathryn Moore’s laboratory and grown in a colony at an NIH animal facility. To assess the TCL (see protocol below) effects in vivo, ~550–600 μL (20 µL per g) of TCL preparation were injected intraperitoneally into control (n = 15) and CD36-deficient (n = 20) mice. Three hours later mice were sacrificed via cervical dislocation. Immediately mice were cut open and perfused using a syringe filled with 10mL of PBS via vena cava inferior, while snipping the portal vein at the same time, to wash out blood from the organs. Blood was collected to prepare plasma samples, organs were harvested and placed into RNA later solution for further RNA isolation and qRT PCR analysis.

### 2.3. Cell Cultures

Human embryonic kidney cells (HEK-293) were obtained from ATCC (cat. # CRL-1573) and were stably transfected to express CD36 (HEK-CD36) using the CD36 pIRES-hrGFP-2a plasmid (Stratagene), followed by selecting cells with the highest GFP expression. Cells were grown in Dulbecco’s modified Eagle’s medium (DMEM) supplemented with 10% fetal calf serum (FCS), 100 IU/mL penicillin, 100 μg/mL streptomycin, and 100 μg/mL G418 at 37 °C in a 5% CO_2_-humidified atmosphere.

Murine *CD36−/−* and *wild-type* (WT) macrophages were isolated from murine bone marrow cells (BMC) obtained from *CD36−/−* mice and control *wild-type* strains, respectively. The macrophages were differentiated by culturing in RPMI-1640 supplemented with 10% fetal calf serum (FCS), in the presence of 10 ng/mL of mouse M-CSF and for 7–10 days.

### 2.4. Total Cellular Lysate and Skeletal Muscle Tissue Lysate Preparation

To obtain total cellular lysate (TCL), HeLa cells (ATCC, cat. # CCL2) cultured to confluency in 150 mm tissue culture dishes were washed three times with ice-cold PBS, scraped, and resuspended in PBS containing a protease and phosphatase inhibitor cocktail. They were then homogenized using a Precellys 24 homogenizer (Bertin Technologies, Montigny Le Bretonneux, France).

To prepare cytosolic and membrane fractions from total cell/tissue lysates (TCL), fresh or frozen mouse skeletal muscle tissue or scraped HeLa cells were washed with ice-cold PBS; cells were pelleted by centrifugation at 2000× *g* for 5 minutes, whereas muscle tissue samples were placed in a pre-chilled glass Petri dish and minced on ice using sharp scissors. Next, cell or tissue samples were resuspended in ice-cold lysis buffer, containing 20 mM Tris–HCl pH 7.4, 5 mM MgCl_2_, 1mM DTT, and protease/phosphatase inhibitor cocktails, incubated on ice for 5 min, and homogenized using a Precellys 24 homogenizer. The homogenates were decanted into centrifuge tubes, maintained on ice for 15 min, vortexed at maximum speed for 15 s, and centrifuged at 1000× *g* for 5 min to remove unbroken cells and *nuclear* debris. Pellets (nuclei + cell debris) were discarded, and the supernatants (total cell lysate, containing plasma membranes + microsomal fraction + cytosol) were collected and used for the preparation of cytosolic (CF) and membrane (MF) fractions. Supernatants were centrifuged at 50,000× *g* for 1 h, and the resulting soluble cytosolic fractions (CF) as well as the pellets (MF), resuspended in equivalent to CF volume of PBS, were used in cell culture studies. We have established that CF and MF prepared in the same manner from HEK293 cells yielded similar results as HeLa-originated preparation when used for the treatment of cultured cells. We chose HeLa cells as a source of cell lysate for our studies because these cells are easier to handle and are more resilient over multiple passages.

The CF preparation that was obtained from one 150 mm culture dish with confluent monolayer, in a final volume of 2 mL, was considered 100% CF. For dose-dependent assays, 3-fold serial dilutions of 10% CF were prepared. For skeletal muscle, 2 mL of CF preparation was obtained from 1 g of fresh muscle tissue.

### 2.5. Total RNA Isolation and qRT-PCR Analysis of Pro-Inflammatory Markers in Murine Tissues

Following 3 hours after the IP injection of the TCL, animals were euthanized, and organs were harvested for further analysis. For RNA isolation, liver and lung tissue samples preserved in RNA-later and stored at −80 °C were homogenized in TRIzol Reagent using a Precellys 24 homogenizer (Bertin Technologies, Montigny-le-Bretonneux, France). RNA was isolated using the PureLink RNA Mini Kit (Thermo Fisher Scientific) after DNase treatment. RNA (2 μg) was reverse transcribed using a TaqMan Reverse Transcriptase Reagent Kit. Real-time qPCR assays were performed with a StepOne Real-Time PCR System (Applied Biosystems, Carlsbad, CA, USA), using 40 ng cDNA per reaction. A list of TaqMan Gene Expression assays used in this study is shown in Table 1. Relative levels of gene expression were measured by the comparative CT method (ΔΔCT) with mouse β-actin or GAPDH genes as reference genes. All gene expression results were analyzed using the 2^−ΔΔCT^ formula and presented as normalized fold changes, compared to corresponding PBS-treated controls.

### 2.6. Statistical Analysis

The data are expressed as the means ± STD for each group. Graphical and statistical analyses were performed using GraphPad Prism, version 7.02 (GraphPad, La Jolla, CA, USA). An unpaired Student’s *t*-test was used to determine the level of statistical significance between sets of data. A *p* value < 0.05 was considered statistically significant. *p* values greater than 0.05 but less than 0.1 were considered a trend toward significance.

## 3. Results

### 3.1. Pro-Inflammatory Responses Induced by the HeLa Cell Cytosol and Skeletal Muscle Cytosol Fractions Are CD36-Dependent

**Figure 1:**
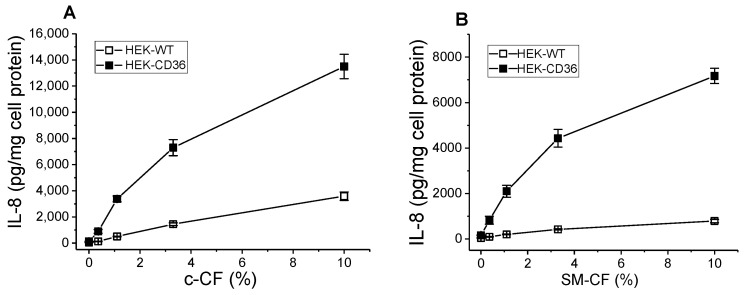
Dose-dependent IL-8 secretion induced by HeLa cell and skeletal muscle CF in WT and CD36-overexpressing HEK293 cells. WT and CD36-overexpressing HEK293 cells were incubated with increasing concentrations of the CF preparations (see Section 2) from HeLa cells, c-CF (**A**) or murine skeletal muscle, SM-CF (**B**) for 20 h. IL-8 levels were quantified in duplicate samples of cell culture supernatants by ELISA. Data represent one of three separate experiments that yielded similar results.

To assess the role of CD36 as a mediator of DAMP-mediated inflammation, we prepared a cell cytosol fraction (c-CF), representing a combination of soluble DAMPs and investigated its effects on pro-inflammatory cytokine IL-8 secretion in WT and CD36-overexpressing HEK293 cells. Following a 20 h incubation of control and CD36-overexpressing cells with gradual dilutions of HeLa cell CF preparation (Figure 1A), we observed a dose-dependent increase in IL-8 secretion, which was markedly (4–5 times) higher in CD36+ cells vs. WT control cells. Additionally, we have tested the cytosol fraction prepared from the skeletal muscle (SM-CF) of normal mice, as an alternative prototype mix of DAMPs, that could be considered a model more closely imitating the release of DAMPs, occurring during the skeletal muscle crush injury. As can be seen in Figure 1 (panel B), after a 20 h incubation this preparation induced a strong dose-dependent inflammatory response in CD36-overexpressing cells, which was significantly (7–8 times) higher compared to the IL-8 response observed in WT cells.

### 3.2. HMGB1- and LPS-Induced IL-8 Secretion in WT and CD36 Expressing HEK293 Cells

**Figure 2:**
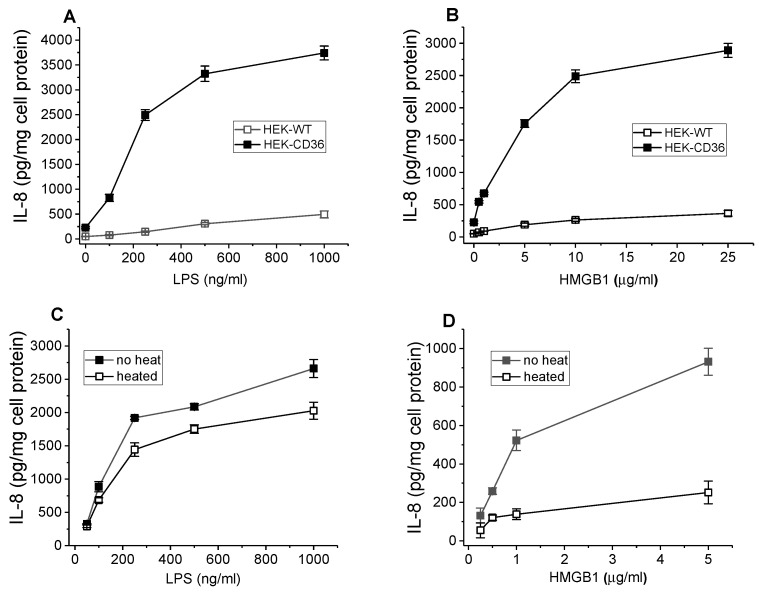
Dose-dependent IL-8 secretion induced by the HMGB1 and LPS in WT and CD36-overexpressing HEK293 cells. Effect of heat treatment on the cytokine-inducing activity of HMGB1 and LPS. WT and CD36-overexpressing HEK293 cells were incubated with increasing concentrations of recombinant HMGB1 (**A**) or LPS (**B**) for 20 h. IL-8 levels were quantified in duplicate samples of cell culture supernatants by ELISA. Data represent one of three separate experiments that yielded similar results. The IL-8 levels were determined using duplicate samples of cell culture supernatants collected after a 20 h incubation of cells with either intact or heat-treated (100 °C for 45 min) HMGB1 (**C**) and LPS (**D**). The data presented are from one of two separate representative experiments.

Thus far, multiple DAMPs have been identified, including HMGB1 which is a critical intracellular protein that regulates gene transcription under normal conditions and acts as an endogenous danger signal through activation of the innate immune system. A previously published report indicated that HMGB1-induced pro-inflammatory signaling in macrophages may be mediated by CD36 [31]. We have assessed the HMGB1-induced inflammatory response in WT and CD36+ cells by measuring IL-8 secretion in cell culture media collected after a 20 h incubation. Recombinant HMGB1 at doses from 0.5 to 25 µg/mL induced up to 10-fold higher levels of IL-8 in CD36+ cells compared to control cells (Figure 2A). Bacterial wall component lipopolysaccharide (LPS), another well-known CD36 ligand used as a positive control, also demonstrated significantly higher IL-8 secretion levels in CD36-overexpressing cells.

LPS contamination could potentially contribute to the observed HMGB1-induced cytokine release. To dissociate the pro-inflammatory activity of LPS and HMGB1 protein, the effects of heat treatment on the cytokine-inducing activity of HMGB1 as well as of LPS were analyzed using CD36-overexpressing HEK293 cells. Heat-induced denaturation for 45 min at 100 °C resulted in a significant reduction (by 70–80%) of the IL-8-inducing activity of HMGB1 (Figure 2C). In contrast, a much smaller reduction (by 25%) of cytokine-inducing activity of the LPS was observed (Figure 2D). Thus, our data indicate that HMGB1-induced cytokine secretion is associated mostly with protein activity rather than LPS contamination.

### 3.3. Pro-Inflammatory Responses Induced by the c-CF, SM-CF, and HMGB1 in CD36 Expressing HEK293 Cells Can Be Blocked by SAHPs

**Figure 3:**
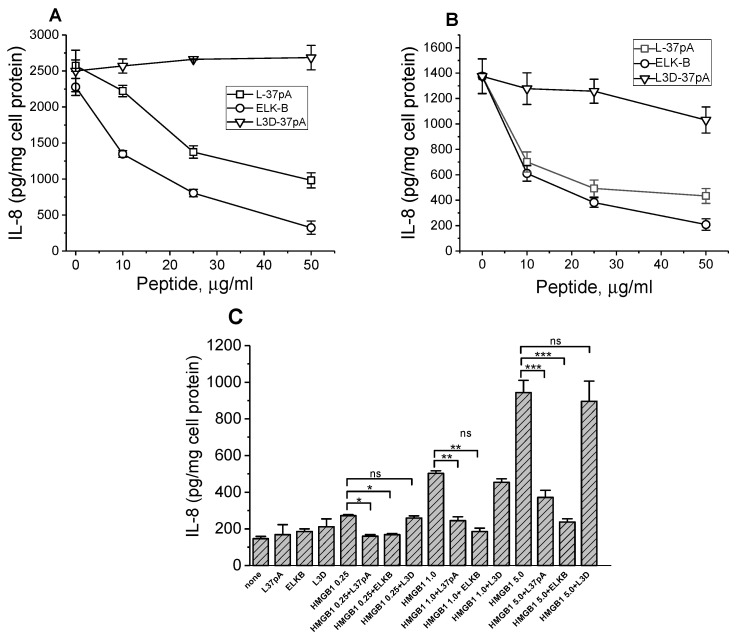
Effects of SAHPs on IL-8 secretion induced by the CF preparations and HMGB1 in CD36-overexpressing HEK293 cells. Cells were preincubated with or without increasing doses of L-37pA, ELK-B, or L3D-37pA for 1 h before a 20 h treatment with 1% c-CF (**A**) or 0.3% SM-CF (**B**). Cells were preincubated for 1 h with or without 10 µg/mL of L-37pA, ELK-B, or L3D-37pA before a 20 h treatment with increasing doses of HMGB1 (0.25, 1, and 5 µg/mL). IL-8 levels were determined in cell culture supernatants in duplicate (**C**). Data are from one of at least two representative experiments. * *p* < 0.05, ** *p* < 0.01, *** *p* < 0.001, and ns—nonsignificant, versus no peptide.

Synthetic amphipathic helical peptides (SAHPs) designed as apolipoprotein A-I mimetics are known to bind to class B scavenger receptors (SR-Bs), SR-BI, SR-BII, and CD36 [8,36,39]. IL-8 secretion induced either by c-CF (Figure 3A) or by SM-CF (Figure 3B) as well as by HMGB1 (Figure 3C) in CD36-overexpressing HEK293 cells were efficiently blocked by the most characterized apoA-I mimetic peptide, L-37pA. Another peptide, ELK-B, previously identified as an SAHP selectively targeting CD36 rather than SR-BI/II, was found to be an even more potent inhibitor of the inflammatory responses induced by both cytosol preparations and HMGB1. The peptide L3D-37pA, containing three D-amino acid substitutions disturbing the amphipathic α-helical motif, was used as a negative control and did not affect IL-8 secretion.

### 3.4. IL-8 Secretion Induced by Histone H3 Is CD36-Dependent and Can Be Reduced by L37pA

**Figure 4:**
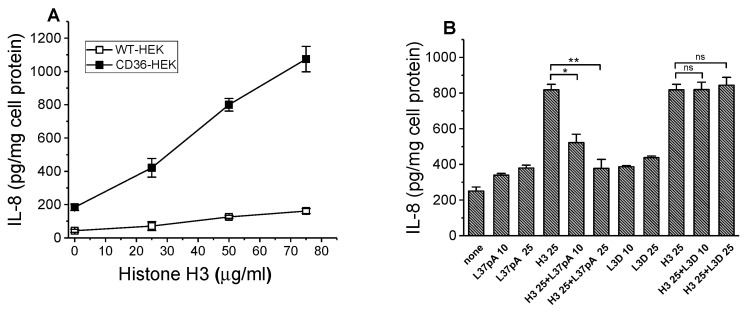
Dose-dependent IL-8 secretion induced by the histone H3 in WT and CD36-overexpressing HEK293 cells. Effect of L-37pA on hH3-induced IL-8 secretion in CD36-HEK293 cells. WT and CD36-overexpressing HEK293 cells were incubated with increasing concentrations of histone H3 for 20 h. IL-8 levels were quantified in duplicate samples of cell culture supernatants by ELISA (**A**). Data represent one of three separate experiments that yielded similar results. CD36-HEK293 cells were preincubated for 1 h with 0, 10 µg/mL, and 25 µg/mL of L-37pA or L3D peptides before a 20 h treatment with 25 µg/mL of histone H3. IL-8 levels were determined in cell culture supernatants in duplicate (**B**). * *p* < 0.05, ** *p* < 0.01, and ns—nonsignificant, versus histone H3 alone. Data are from one of at least two representative experiments.

Histones are a family of small, positively charged proteins that are found in eukaryotic cell nuclei and are known to bind DNA and regulate gene expressions. Following tissue damage, histones can be released into the extracellular space by activated or damaged cells and act as cytotoxic DAMP proteins by activating PRR and promoting a pro-inflammatory cytokine response. In this study, we evaluated the CD36 contribution to the pro-inflammatory response induced by the histone H3 (hH3), one of the four core histones. Our data demonstrate a dose-dependent increase in IL-8 secretion in CD36-overexpressing cells following a 20 h incubation with a range of hH3 concentrations, with significantly (6–8 times) higher inflammatory responses, compared to control WT cells (Figure 4A). Importantly, hH3-induced CD36-dependent IL-8 secretion was efficiently reduced by the CD36 antagonist, L37-pA peptide (Figure 4B).

### 3.5. Pro-Inflammatory Responses Induced by Multiple Other DAMPs Are Markedly Higher in CD36-Overexpressing vs. Wild Type HEK293 Cells

**Figure 5:**
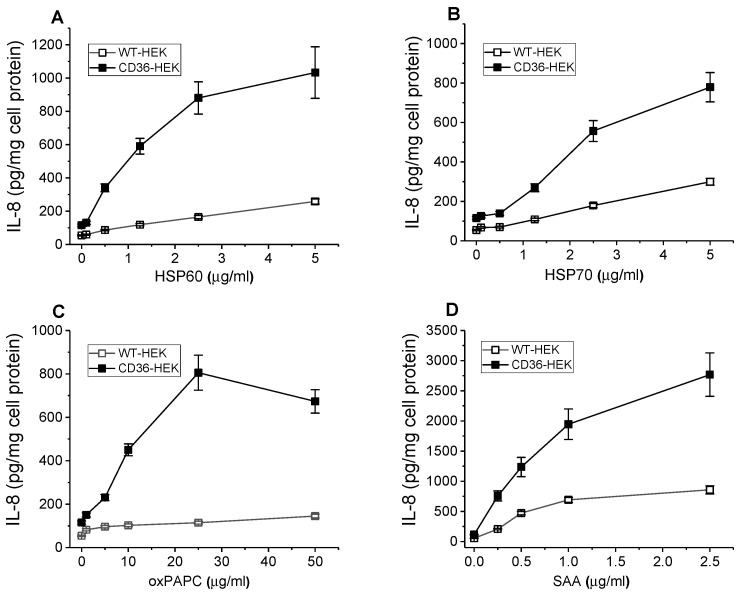
DAMPs-induced dose-dependent pro-inflammatory responses in WT and CD36-overexpressing HEK293 cells. WT and CD36-overexpressing HEK293 cells were incubated with increasing concentrations of HSP60 (**A**), HSP70 (**B**), oxPAPC (**C**), and SAA (**D**) for 20 h. IL-8 levels were quantified in duplicate samples of cell culture supernatants by ELISA. Data represent one of two separate experiments that yielded similar results.

To further evaluate CD36’s role in pro-inflammatory signaling induced by other known DAMPs, we compared the effects of heat shock proteins, HSP60 and HSP70 on IL-8 release by control WT and CD36-overexpressing HEK293 cells following a 20 h incubation with these proteins. Both HSPs used in the range of concentrations from 0.25 to 5 µg/mL induced strong and dose-dependent responses that were significantly higher (~4–5 times) in CD36-overexpressing cells vs. control cells (Figure 5A,B).

Endogenous oxidized phospholipids, oxPAPC (oxidized 1-palmitoyl-2-arachidonoyl-*sn*-glycero-3-phosphorylcholine) are produced during tissue stress and are responsible for sustaining inflammatory responses in both immune and non-immune cells. Earlier studies demonstrated CD36 as a receptor mediating binding and uptake of oxPAPC in macrophages, promoting foam cell formation during atherosclerosis [40,41]. We have compared the effect of an oxPAPC preparation on pro-inflammatory cytokine secretion in cultured WT and CD36-overexpressing HEK293 cells. Our data presented in Figure 5C indicate that the stimulatory effect of oxPAPC on IL-8 secretion was strongly dependent on CD36 as cytokine levels induced by oxPAPC in HEK-CD36 were markedly (up to 7–8 times) higher compared to those detected in HEK-WT cells. Additionally, Figure 5D demonstrates a similar magnitude difference between the pro-inflammatory responses of WT and CD36-overexpressing cells, induced by the serum amyloid A (SAA), another well-known DAMP, and previously reported ligand of CD36 [8].

### 3.6. DAMP-Induced IL-6 Secretion Is Reduced in BMDM from CD36−/− Mice Compared to Wild Type Mice

**Figure 6:**
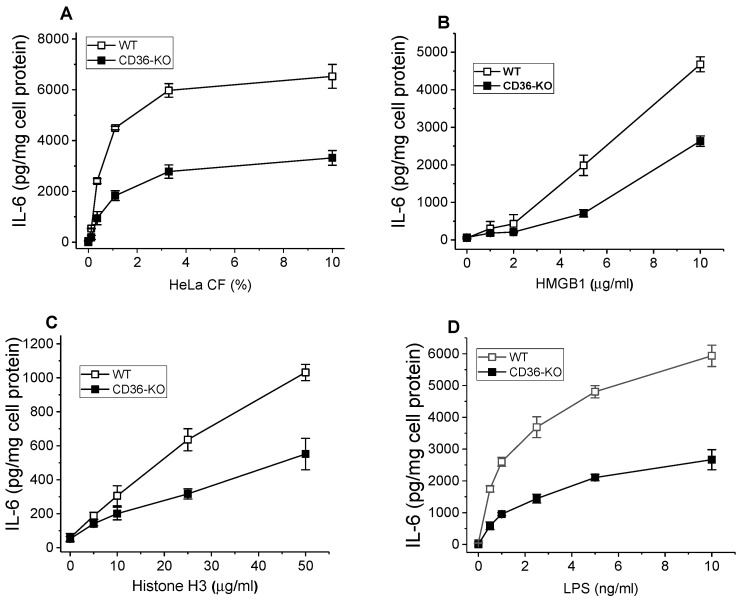
Pro-inflammatory responses induced by the various DAMPs in BMDM from WT and CD36-knockout mice. BMDM isolated from WT and CD36−/− mice were incubated with increasing doses of cCF (**A**), HMGB1 (**B**), histone H3 (**C**), and LPS (**D**) for 20 h. IL-6 levels were quantified in duplicate samples of cell culture supernatants by ELISA. Data represent one of two separate experiments that yielded similar results.

SR-Bs, and CD36 in particular, are abundantly expressed in phagocytic cells, including macrophages, where along with TLRs they play a critical role as PRR, recognizing multiple PAMPs and DAMPs that via activation of signaling pathways regulate innate immune responses. To assess the CD36 contribution to DAMP-induced pro-inflammatory signaling in macrophages, we compared levels of IL-6 release in BMDM from wild-type and CD36-knockout mice, following a 20 h stimulation with CF preparation, HMGB1, and histone H3. As is shown in Figure 6A, IL-6 secretion was markedly reduced (~40–50%) in CD36-deficient macrophages, compared to the response in cells from normal mice. Likewise, the DAMPs, HMGB1, and hH3, also induced lower cytokine responses in CD36−/− vs. wild type BMDM, by 45–60% and ~50%, respectively (Figure 6B,C), mostly observed with the higher doses of these ligands. LPS, a well-known PAMP and SR-B ligand, was used as a positive control and induced a ~35–50% lower pro-inflammatory IL-6 response in CD36-deficient macrophages when compared to wild-type control cells (Figure 6D).

### 3.7. Effects of MAPKs and Src Family Kinase Inhibitors on CF-Induced IL6 Secretion in Normal and CD36−/− Murine BMDM

**Figure 7:**
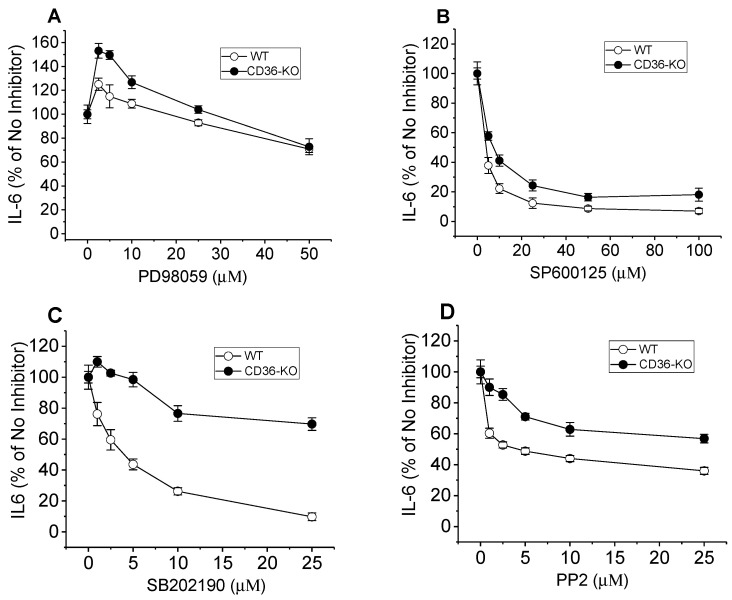
Effects of signaling pathways Inhibitors on CF-induced IL-6 secretion in BMDM from WT and CD36-knockout mice. BMDM isolated from WT and CD36−/− were pre-incubated for 1 h with increasing doses of PD98059 (**A**), SP600125 (**B**), SB202190 (**C**), or PP2 (**D**). Following pre-incubation, cells were incubated with CF preparation for the next 20 h. IL-6 levels were quantified in duplicate samples of cell culture supernatants by ELISA. Data represent one of two separate experiments that yielded similar results.

To analyze the potential mechanisms of CD36-dependent cytokine secretion induced by the CF preparation, we tested pharmacological inhibitors of major known signaling cascades, using normal and CD36-deficient murine macrophages. Cell treatment with the specific MAPK inhibitors—PD98059, SP600125, or SB201190—that selectively block MEK1, the upstream kinase of ERK1/2, JNK, and p38 kinase activity, respectively, led to variable reductions in CF-induced levels of IL-6 in cell culture media (Figure 7). While the MEK1 inhibitor had a barely noticeable effect and only at its highest dose in both cell types (Figure 7A), the JNK inhibitor appeared to be an equally potent blocker of CF-induced IL-6 secretion in BMDM from wild-type and CD36-KO mice (Figure 7B). At the same time, the p38 inhibitor efficiently blocked (by >80%) IL-6 secretion in normal macrophages, while its effect was significantly lower (~30% inhibition) in CD36-deficient cells (Figure 7C). Cell treatment with PP2, an inhibitor of protein tyrosine kinases of the Src family, known as upstream kinases of MAPKs activation, resulted in moderate blocking of CF-induced IL6 secretion (by about 50–60%) in normal BMDM and to a lesser extent (by 30–40%) in CD36-KO cells (Figure 7D).

### 3.8. Acute Inflammatory Responses in WT and CD36−/− Mice Accessed by the Liver and Lung Pro-Inflammatory Gene Expression 3 h After the IP Injection of TCL

**Figure 8:**
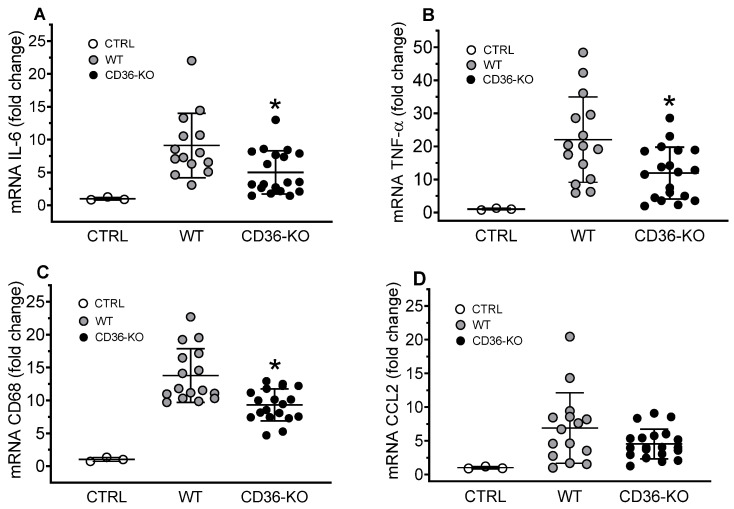
Hepatic gene expression of inflammatory markers in WT and CD36-knockout mice IP injected with a TCL preparation. Livers were collected for mRNA extraction and qRT-PCR assay as described in Materials and Methods. Expression levels of IL-6 (**A**), TNF-α (**B**), CD68 (**C**), and CCL2 (**D**) were normalized by GAPDH expression and are presented as the fold change relative to the PBS-treated WT control mice. Values shown are the means ± STD (n = 12–15 for the WT group, n = 20 for the CD36-KO group). * *p* < 0.05, versus WT TCL-treated mice.

To explore the role of CD36 as a contributor to DAMP-induced pro-inflammatory response in vivo, we used an intraperitoneal injection of total cell lysate (TCL) preparation into wild-type and CD36-knockout mice. We have found that both cytosol (CF) and membrane (MF) cellular fractions were potent inducers of IL-8 production in CD36-overexpressing HEK293 (Supplemental Figure S1). To maximize the pro-inflammatory response, for in vivo experiments instead of using isolated CF we used total cell lysate (TCL), containing (1) soluble DAMPs of CF, (2) plasma membrane and microsome fraction (MF), and (3) nuclear fraction. To evaluate local tissue inflammation, pro-inflammatory gene expression levels were measured in the livers and lungs of wild-type and CD36-KO mice subjected to an acute (3 h) IP injection of TCL. The expression levels of pro-inflammatory markers, which included IL-6, TNF-α, and CD68 were statistically lower, by 45%, 46%, and 33%, respectively, (Figure 8, panels B, C, and D) in the livers of CD36−/− mice, when compared to wild type mice.

**Figure 9:**
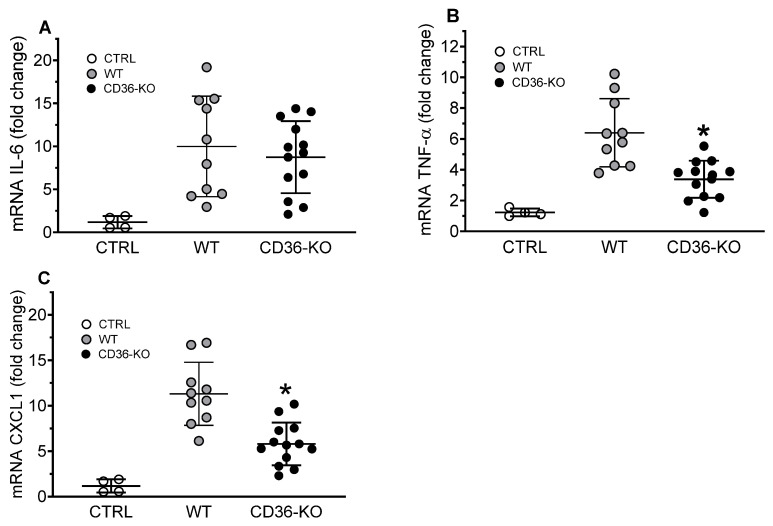
Pulmonary gene expression of inflammatory markers in WT and CD36-knockout mice IP injected with a TCL preparation. Lungs were collected for mRNA extraction and qRT-PCR assay as described in Materials and Methods. Expression levels of IL-6 (**A**), TNF-α (**B**), and CXCL1 (**C**) were normalized by GAPDH expression and are presented as the fold change relative to PBS-treated WT control mice. Values shown are the means ± STD (n = 10 for the WT group, n = 14 for the CD36-KO group). * *p* < 0.05, versus WT TCL-treated mice.

In the lungs, the expression levels of cytokines TNFα (Figure 9B) and CXCL1 (Figure 9C) were found to be reduced by approximately 50% in CD36-deficient vs. wild-type mice, with no statistically significant difference in IL-6 expression between the mice groups (Figure 9A).

## 4. Discussion

Ischemia–reperfusion, crush syndrome, surgical procedures, blood loss, or massive transfusion, hypoxemia, burns, and other traumas and injuries can all induce tissue damage, followed by the release of DAMPs from the damaged cells/tissues into the extracellular environment and blood circulation with subsequent activation of innate and adaptive immunity. Excessive production of DAMPs or their failed clearance may lead to chronic inflammation and delayed inflammation resolution. DAMPs are heterogeneous in their origin and function; however, they all induce sterile inflammation, that involves cytokine release, neutrophil recruitment, and the induction of T-cell immunity [42]. Activation of the innate immune response is initiated as a result of DAMP interaction with the PRRs, which include Toll-like receptors (TLRs) and scavenger receptors, as well as non-PRRs, such as RAGE [43]. TLRs are integral membrane proteins known to play a key role in the recognition of DAMPs, in particular, nucleic acids, HMGB1, and HSPs [14,25,44]. The role of class B scavenger receptors as PRRs for DAMPs is not fully elucidated, although several reports identified CD36 as a co-receptor, facilitating DAMP-induced TLRs’ downstream signaling [7,45]. A study by Stewart et al. [46] demonstrated that binding of oxidized LDL and amyloid-beta recognized endogenous DAMPs and known CD36 ligands, initiate proximal signaling required for TLR4-TLR6-heterodimer assembly and subsequent inflammatory response, thus emphasizing CD36’s role as a TLRs accessory molecule. The findings of another study also indicate that CD36 can contribute to TLRs-mediated signaling by facilitating the association of bacterial LTA with TLR2/TLR6 heterodimers on the cell surface [47]. This cooperative relationship between TLRs and CD36 plays a particularly important role in the reticuloendothelial cells, such as macrophages, that represent the primary defense against microbial pathogens. Macrophages are known to express high levels of TLRs, TLR2 [48], and TLR4 [49], particularly, as well as class B scavenger receptors, including CD36 (Supplementary Figure S2, panel C). On the other hand, the HEK293 epithelial cell line is characterized by non-detectable levels of TLR2 and TLR4 [50,51,52] and very low expression of class B scavenger receptors, CD36 and SR-BI (Supplementary Figure S2, panels A and B), and therefore, represents a suitable control for CD36-overexpressing cells used in this study. Findings of our previous studies utilizing HEK293 CD36-overexpressing cells, as well as another recent report, indicate that SR-Bs, and CD36 in particular, could be directly involved in the recognition and mediating of pro-inflammatory signaling of several DAMPs, including SAA [3], HSP60 [6], and HMGB1 [31].

The objective of the current study was to further investigate CD36’s direct role as a DAMP receptor capable of mediating sterile inflammation induced by the various individual DAMPs as well as by the prototype of their mixture in the form of cellular /tissue lysate. For in vitro studies, using human epithelial cells overexpressing CD36, we evaluated CD36-dependent pro-inflammatory effects of either cell cytosol or skeletal muscle cytosol fractions, representing a mixture of multiple soluble DAMPs. For in vivo studies to maximize the inflammatory response induced by the DAMPs, the crude total cell homogenate, containing a combination of DAMPs from different intracellular sources, such as mitochondria, plasma membranes, ER, and microsomal membranes, was used for IP injection to control and CD36-knockout mice.

HMGB1 is a nuclear protein that regulates gene transcription under normal conditions and acts as an endogenous danger signal through activation of the innate immune system, when released from damaged various cells, including monocytes, macrophages, dendritic cells, natural killer cells, endothelial cells, and tumor cells. There is evidence that HMGB1 is critically important for both triggering and resolving inflammation, which follows infection or trauma. A previous study [31] demonstrated that HMGB1-activated Erk and Akt signaling in BMDM from CD36-deficient mice is markedly impaired, suggesting a direct role of CD36 as a mediator of the HMGB1 effect on macrophages. In our study using 2 different cell models, CD36+ epithelial cells and CD36−/− macrophages, we demonstrated that HMGB1-induced pro-inflammatory cytokine responses were CD36-dependent in both cases.

Heat shock proteins are present in most cells, and normally function as chaperones that assist with protein folding and biosynthetic pathways [53], but extracellular HSPs, the products of cellular necrosis, play an important role in response to cell damage and stress stimuli and are known to induce inflammation mostly through the activation of TLR2, TLR4, and CD91 [24,54,55,56]. Our earlier study [6] demonstrated that class B scavenger receptors, including CD36, are receptors recognizing and mediating pro-inflammatory signaling induced by the recombinant chaperonin 60 (HSP60). Here, our data demonstrate both HSP60 and HSP70 as potent inducers of pro-inflammatory response in a CD36-dependent manner, further underscoring CD36’s role as a PRR recognizing various endogenous danger signals.

Histones are highly basic DNA-bound proteins that are found in the nuclei of the eukaryotic cells and regulate gene expression. Besides their intranuclear function, histones can act as DAMPs when released into the extracellular space during cell death processes, such as necrosis or apoptosis, exhibiting significant toxic or pro-inflammatory activity [57,58]. According to previously reported data, administration of exogenous histones to animals leads to systemic inflammation and toxic responses through activating Toll-like receptors and inflammasome pathways [59,60,61]. However, the evidence of the histones’ role as DAMPs as well as of the role of other PRRs, besides TLRs mediating their pro-inflammatory effects, is limited. Here, we evaluated one of four core histones, histone H3 (hH3), and we have found that CD36 recognizes hH3 and mediates its pro-inflammatory signaling. Our data, presented in Figure 4A and Figure 6C, demonstrate that hH3-induced cytokine response in HEK293 cells and murine BMDM is CD36-dependent. Moreover, we demonstrated that the hH3-induced IL-8 secretion in CD36-overexpressing HEK 293 cells could be blocked by L37pA, a known SR-B binding peptide (Figure 4B), further supporting CD36’s role as a novel receptor for hH3.

Reactive oxygen species generated during inflammation may result in the oxidation of host phospholipids. Endogenous oxidized phospholipids (oxPL) are produced during tissue stress or injury and are responsible for sustaining inflammatory responses in immune and non-immune cells. Strong pro-inflammatory activities of oxPLs, reported by multiple studies, are mediated by their interaction with a variety of different PRRs of the innate immune system. In particular, OxPLs are directly recognized by several TLRs [62,63,64], scavenger receptors [65,66], as well as by soluble PRRs such as C-reactive protein [67]. Although oxPLs, such as oxPAPC (1-palmitoyl-2-arachidonyl-*sn*-glycero-3-phosphorylcholine), are a well-known modulator of inflammatory responses, their precise role remains controversial and, apparently, context-dependent, as pro-inflammatory and anti-inflammatory effects of oxPAPC have been reported [68]. OxPAPC can function as a TLR4 agonist inducing expression of interleukin IL-6, IL-8, and MCP-1, and promoting adhesion of monocytes [69,70]. Another study demonstrated oxPAPC as an inhibitor of Toll-like receptor signaling induced by bacterial lipopeptide or lipopolysaccharide (LPS). It was previously reported that many of the effects of OxPL are mediated by its interaction with CD36. The binding and uptake of oxPAPC by CD36 on macrophages were demonstrated to activate signaling pathways that promoted foam cell formation during atherosclerosis [68,71]. One of the studies identified a novel family of oxidized phosphatidylcholines (oxPC_CD36_) that serve as highly specific ligands for scavenger receptor CD36 [72]. These oxPC_CD36_ accumulate in vivo and mediate macrophage foam cell formation as well as promote platelet hyper-reactivity in hyperlipidemia via CD36 [66,73]. Our data, which demonstrated markedly higher pro-inflammatory cytokine response induced by the oxPAPC in cells with CD36 overexpression compared to control cells, provide further evidence of CD36’s role as a PRR for oxPL and an important mediator of their inflammatory signaling.

Experimental evidence from earlier studies strongly suggests that, despite having very short intracellular tails, CD36 can function as a signal transduction receptor that initiates a signaling cascade upon ligand binding. Data from several research groups obtained using various cellular systems have linked CD36 signaling function to the recruitment of Src family kinases and activation of specific mitogen-activated protein (MAP) kinases. Moore et al. [74] have demonstrated that binding of the fibrillar amyloid peptide, β-amyloid, to CD36 initiates a pro-inflammatory signaling cascade, involving an Src kinase family member, Fyn, and p44/42 MAP kinase in microglial and other tissue macrophages. In microvascular endothelial cells, thrombospondin-1 induces a CD36-dependent antiangiogenic, proapoptotic signal via activation of Fyn, caspase-3, and p38 MAP kinase. [75]. It was also demonstrated that macrophage exposure to oxLDL leads to the recruitment of Lyn and activation of c-Jun N-terminal kinase (JNK) in a CD36-dependent manner [71]. Earlier findings of our lab demonstrated that CD36 binds serum amyloid A (SAA) and mediates SAA-induced pro-inflammatory, signaling predominantly through JNK- and ERK1/2-mediated signaling pathways [3].

In this study, using pharmacological inhibitors of major signaling pathways, we attempted to assess potential downstream signaling effectors involved in the CD36-dependent DAMP-induced inflammatory responses using normal and CD36-deficient macrophages. We have found that the blocking effect of JNK inhibitor on CF-induced cytokine secretion was very prominent (80–90% of inhibition), but not solely associated with the CD36-dependent pathway, as it was similar in both types of macrophages, with or without CD36 expression. At the same time, the blocking effect of a p38 inhibitor was markedly stronger (up to 80%) in normal macrophages vs. CD36-deficient cells (up to 30%), suggesting an involvement of the p38 signaling pathway in CD36-mediated DAMP-induced inflammatory response.

Following IP injection of TCL preparation, we have found markedly elevated expression levels of several pro-inflammatory markers in the liver and lung tissues of both, normal and CD36-deficient mice. However, TCL-induced pro-inflammatory response was approximately two times higher in wild-type vs. CD36-knockout mice. These tissue-specific differences in the expression of pro-inflammatory cytokines between the mice groups suggest a CD36 contribution to TCL-induced hepatic and pulmonary inflammation.

Overall, our data demonstrate that the pro-inflammatory activity of several known DAMPs, used either as an individual stimulus or as a mixed preparation of DAMPs, originating from different cellular sources, can be mediated through the CD36 receptor, thus representing a promising target for therapeutic intervention.

## 5. Conclusions

In conclusion, establishing CD36 as an important contributor to DAMP-induced inflammation expands its previously widely known role as a PRR for PAMPs. These results identify CD36 as a potential target during some known traumatic conditions, associated with mechanical tissue injuries, such as crush injury, as well as other conditions, including brain ischemia, hemorrhagic shock, hepatic necrosis, sickle cell anemia, that involve sterile inflammation and tissue damage induced by the increased release of DAMPs. Additionally, our data demonstrating that SAHPs, known to be bound by CD36, could efficiently reduce DAMP-induced inflammation, further underscore the potential therapeutic importance of blocking CD36 to treat excessive inflammation that could be detrimental to the host. However, further in vivo studies involving testing other CD36 pharmacological inhibitors, such as small molecules and synthetic peptides on CD36-mediated inflammation, are needed to confirm this receptor as a suitable candidate for therapeutic implications aimed at alleviation and/or treatment of inflammatory conditions associated with the excessive DAMPs production.

## Figures and Tables

**Table 1:** TaqMan gene expression assays used in the study.

Species	Gene Name	Gene Symbol	Thermo Fisher Scientific ID Number
Mouse	Interleukin 6	Il6	Mm00446190_m1
Mouse	Chemokine (C-C motif) ligand 2	Ccl2	Mm00441242_m1
Mouse	Tumor necrosis factor	Tnfa	Mm00443258_m1
Mouse	Glyceraldehyde-3-phosphate dehydrogenase	Gapdh	Mm03302249_g1

## Data Availability

The raw data supporting the conclusions of this article will be made available by the authors upon request.

## Supplementary Materials

The following supporting information can be downloaded at https://www.mdpi.com/article/10.3390/cells13231992/s1, Figure S1: IL-8 secretion levels induced by the cytosol (CF) and membrane (MF) cellular fractions in CD36-overexpressing HEK293 cells; Figure S2: Western Blotting analyses of CD36 and SRBI expression in WT and SRB-expressing clones of HEK293 cells, and mCD36 expression in murine bone-marrow-derived macrophages.
