# Supplementary material for: Class B Scavenger Receptor CD36 as a Potential Therapeutic Target in Inflammation Induced by Danger-Associated Molecular Patterns
Source: Cells. 2024 Dec 3;13(23):1992. doi: 10.3390/cells13231992 (PMC11640246; doi:10.3390/cells13231992)
Supplement: Supplementary file 1 [file cells-13-01992-s001.zip › cells-3313123-supplementary.pdf]

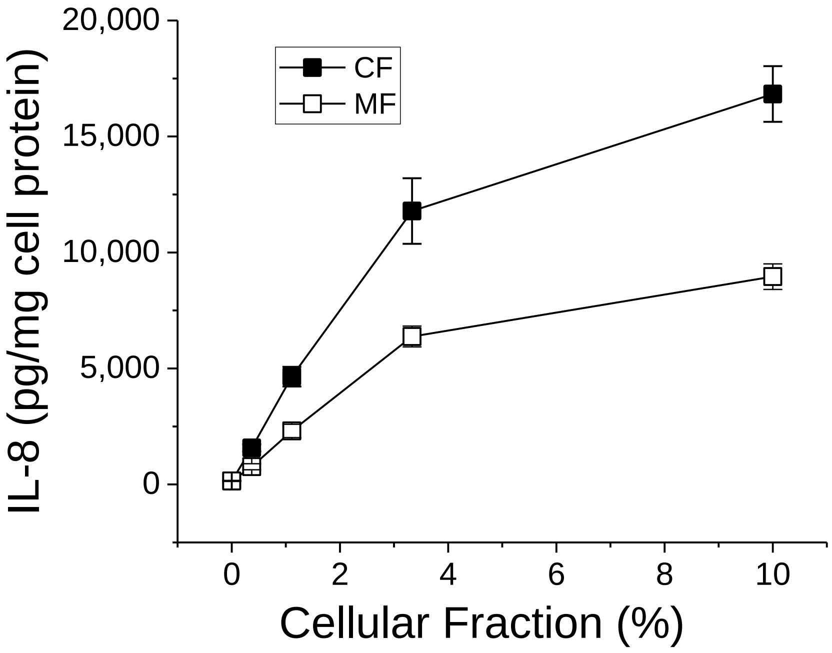

Figure S1: IL-8 secretion levels induced by the cytosol (CF) and membrane (MF) cellular fraction in CD36-overexpressing HEK293. CD36-overexpressing HEK293 were incubated with increasing concentrations of CF or MF preparations (see Material and Methods) for 20 h. IL-8 levels were quantified in duplicate samples of cell culture supernatants by ELISA. Data represent one of two separate experiments that yielded similar results.

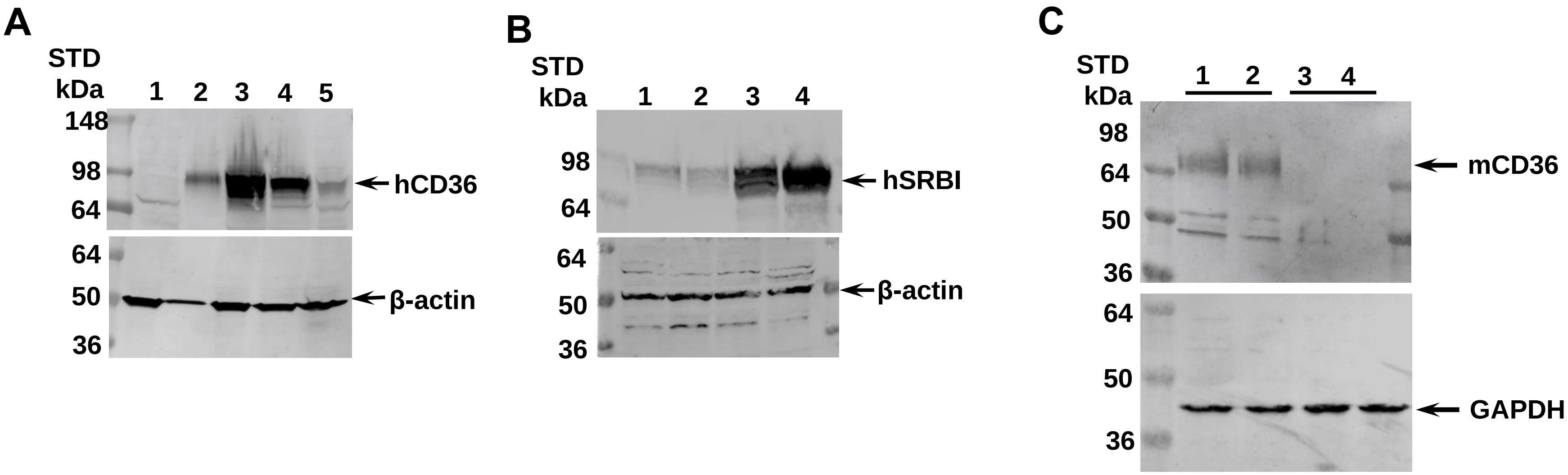

Figure S2: Western Blotting analyses of CD36 and SRBI expression in WT and SRB-expressing clones of HEK293 cells, and mCD36 expression in murine bone marrow-derived macrophages. **A**. CD36 expression in HEK 293 cells: lane 1-HEK293 wild type; lanes 2-4-clones of CD36-overexpressing HEK293 cells; lane 5-A549 cell line. **B**. SRBI expression in HEK293 cells: lanes 1-2-HEK293 wild type; lanes 3,4-clones of SRBI-overexpressing HEK293 cells. **C.** CD36 expression in BMDM from wild type and CD36-KO mice. hCD36 and hSRBI protein expressions were detected using anti-hCD36 antibody (R&D Systems, cat. # AF1955) and anti-hSRBI antibody (BD Biosciences, cat. # 610882), respectively. mCD36 was detected by using anti-mCD36 antibody (R&D Systems, cat. # AR2519). Protein expression of β-actin or GAPDH were measured as loading controls using anti-β-actin polyclonal antibody (Sigma Aldrich, cat. #A2103) or anti-GAPDH monoclonal antibody (Invitrogen, cat. # MA5-15738), respectively.
